# The glycopatterns of *Pseudomonas aeruginosa* as a potential biomarker for its carbapenem resistance

**DOI:** 10.1128/spectrum.02001-23

**Published:** 2023-10-20

**Authors:** Jing Dang, Jian Shu, Ruiying Wang, Hanjie Yu, Zhuo Chen, Wenbo Yan, Bingxiang Zhao, Li Ding, Yuzi Wang, Huizheng Hu, Zheng Li

**Affiliations:** 1 Laboratory of Functional Glycomics, College of Life Sciences, Northwest University, Xi'an, Shaanxi, China; 2 Hospital of Shaanxi Nuclear Industry, Xianyang, Shaanxi, China; University of California, San Diego, La Jolla, California, USA

**Keywords:** carbapenem-resistant *P. aeruginosa *(CRPA), lectin microarrays, glycan structures, bacterial cell surface, machine learning

## Abstract

Multidrug-resistant (MDR) *Pseudomonas aeruginosa* infections pose a significant challenge to effective treatment. Although carbapenems were once considered the primary therapeutic option for MDR *P. aeruginosa*, the clinical use of these antibiotics has become increasingly limited, and the exploration of alternative antimicrobial strategies remains necessary. Bacterial surface glycans are critical in response to antibiotics and represent an attractive therapeutic target. However, the understanding of the role of glycan structures in bacterial resistance is currently quite limited. In this study, we used lectin microarrays to analyze the differences in glycan alterations between 53 drug-sensitive *P. aeruginosa* (DSPA) strains and 57 carbapenem-resistant *P. aeruginosa* (CRPA) strains obtained from clinical isolates, with the goal of identifying important glycopatterns associated with carbapenem resistance. The results revealed significant differences in the expression levels of glycan structures [e.g., Fucα1–6GlcNAc, α-d-Man, and Fucα1–3(Galβ1–4)GlcNAc] recognized by 20 lectins (e.g., LCA, PSA, and AAL) on the bacterial surface. Furthermore, we applied *K*-fold cross-validation to determine the optimal parameters and constructed the DSPA and CRPA models using gradient boosting decision tree (GBDT) algorithm. The GBDT model presented the best performance in an independent test cohort, demonstrating that the screened glycopatterns could serve as potential biomarkers for differentiating bacterial resistance. Our study is the first to apply lectin microarrays to monitor carbapenem resistance in clinical *P. aeruginosa* isolates, and we hope that the methodology used in this work will serve as an important tool for exploring the association of bacterial resistance with glycosylation mechanisms.

**IMPORTANCE:**

Bacterial surface glycans are an attractive therapeutic target in response to antibiotics; however, current knowledge of the corresponding mechanisms is rather limited. Antimicrobial susceptibility testing, genome sequencing, and MALDI-TOF MS, commonly used in recent years to analyze bacterial resistance, are unable to rapidly and efficiently establish associations between glycans and resistance. The discovery of new antimicrobial strategies still requires the introduction of promising analytical methods. In this study, we applied lectin microarray technology and a machine-learning model to screen for important glycan structures associated with carbapenem-resistant *P. aeruginosa*. This work highlights that specific glycopatterns can be important biomarkers associated with bacterial antibiotic resistance, which promises to provide a rapid entry point for exploring new resistance mechanisms in pathogens.

## INTRODUCTION

Multidrug-resistant (MDR) bacterial strains are becoming increasingly prevalent, particularly *Pseudomonas aeruginosa*, the most common pathogenic organism in human clinical isolates (1). *P. aeruginosa* is an adaptable opportunistic pathogen capable of infecting various organs, including the respiratory, vascular system, urinary tract, and central nervous system, among others, causing significant morbidity and mortality (1), and is associated with prolonged hospital stays and increased health care costs (2). The adverse clinical outcomes associated with *P. aeruginosa* infection are largely due to its multiple drug resistance, which limits effective antibiotic treatment options and makes eradicating it increasingly difficult (2). Carbapenems used to be the most important therapeutic option against MDR *P. aeruginosa* (3). However, in recent years, carbapenem-resistant strains have become widespread and continue to expand in clinical practice (4, 5). The World Health Organization has listed carbapenem-resistant *P. aeruginosa* (CRPA) as one of the most serious pathogenic threats to humans, and addressing it has become an important issue in public health (6, 7). Further exploration of new antimicrobial strategies continues to be necessary.

Protein glycosylation is a ubiquitous post-translational modification that polymerizes or attaches carbohydrates to target macromolecules (proteins or lipids) and regulates many important biological processes. In bacteria, most glycosylation products are located on the cell surface, including oligosaccharides and polysaccharides such as lipopolysaccharides (LPS) and capsular polysaccharides (CPS), as well as glycoproteins such as pili, flagella, adhesin, self-transporter, and efflux pump (8). The glycosylation on the bacterial cell surface not only plays a crucial role in colonization (9 – 12), adhesion (13 – 18), symbiosis (10, 11), virulence factors (19), pathogenesis (20 – 22), cell-ce ll interactions, and immune evasion (10, 23 – 26) but also contributes directly to bacterial resistance (19, 27 – 33). Biofilm formation enables bacteria to adapt to the microenvironment under antibiotic selection pressure and to produce persister cells to enhance resistance. Biofilms are known to be complex entities consisting of bacterial cells aggregated in a secretory matrix containing polysaccharides, usually adhering to the surface of the organism (34, 35). Defective lipoprotein mannosylation alters the permeability of the cell envelope of *Mycobacteria abscessus*, making it more susceptible to macromolecular compounds acting on the peptidoglycan layer and to antibiotics such as penicillin G, ampicillin, and vancomycin (36). In *Campylobacter jejuni*, the CmeABC complex is a major multidrug efflux pump that is resistant to various antibiotics. When the *N*-glycosylation of CmeA alone was abolished, the function of the entire CmeABC efflux pump was impaired, resulting in a significant increase in sensitivity to azithromycin (37). These findings reflect the critical importance of bacterial surface glycosylation in response to antimicrobial agents, making it an attractive therapeutic target (8). However, compared to other biological processes, the direct impact of glycosylation on the mechanism of bacterial resistance is poorly understood.

Lectin is a non-immunogenic glycan-binding protein that recognizes glycan epitopes of free carbohydrates or glycoproteins with high specificity and is a primary means of glycosylation research. When adapted to microarrays, lectins can analyze a multiplicity of distinct binding glycan patterns and, thus, provide information on the carbohydrate composition of the sample (38, 39). Lectin microarrays have been developed and applied to analyze the glycosylation profiles of different bacterial species, allowing differentiation between given bacterial strains and monitoring changes in sugar signaling associated with environmental conditions (40). For instance, Hsu et al. used a microarray containing 21 lectins to observe significant differences in lectin-binding patterns and intensities between two closely related *Escherichia coli* strains, indicating the presence of different surface glycan structural libraries (41). The invasiveness of *E. coli* RS218 is known to be growth-dependent, with a general decrease in the positive signal intensity observed for 10 lectins when moving from exponential phase to stationary phase, suggesting a potential association between glycosylation and invasion (41). These results demonstrate that lectin microarray can differentiate between *E. coli* strains and monitor the dynamics of cell surface glycans. Furthermore, lectin microarrays have been used to differentiate between *Lactobacillus casei/paracasei* strains that could not be distinguished from each other by 16S ribosomal RNA gene sequencing. By using microarrays containing 44 lectins, half of the 16 strains were bound by only one or two lectins, while the rest were recognized by multiple lectins with different carbohydrate-binding specificity, indicating the diversity of glycan structures (42). The assay allows strains to be differentiated while providing information on the carbohydrates as determinants available for recognition on the bacterial surface. Additionally, lectin microarrays have been applied to analyze the glycosylation profiles of different bacteria and to monitor changes in glycoforms associated with changes in culture conditions. Two clinical isolates of *C. jejuni* were determined using microarrays printed with a set of 41 lectins. When cultured at 42°C, a significantly reduced binding signal for lectins specifically recognizing Gal, lactose[Galβ(1–4)Glc], or GlcNAc was observed in strain 81116. These glycans were present in the LPS-like structure of the strain, indicating reduced or altered expression of the LPS structure, whereas when cultured at 37°C, the cell surface structure was mainly recognized as an LPS-like molecule. In contrast, the hypervirulent strain 81–176 grown at 42°C showed only minor changes in the binding intensity of a fraction of the lectins, implying that the glycan structures that could be recognized were relatively constant; whereas at 37°C incubation, the surface CPS and lipooligosaccharides, which are known to play different roles in the adhesion and invasion of epithelial cells and evasion of the immune system, were better recognized by lectin microarray (43).

In this study, we used lectin microarrays to analyze the differences in the expression levels of glycan structures between 110 clinical isolates of DSPA (DSPA = 53 strains) and CRPA (CRPA = 57 strains). The results showed that the expression levels of multiple glycan structures on the bacterial cell surface recognized by lectins differed significantly between the two groups. Subsequently, we constructed a machine-learning model using a gradient boosting decision tree (GBDT) algorithm in an attempt to identify crucial glycopatterns that could serve as a potential biomarker for the differentiation of clinical DSPA and CRPA. We assessed the effectiveness of the classifier model based on a series of performance parameters, which also exhibited the feasibility of employing the high-throughput technology, lectin microarrays, to identify antibiotic resistance of clinical infectious pathogen. We hope that the methodology used in this work will serve as an important tool for establishing the association of bacterial resistance with glycosylation mechanisms.

## RESULTS

### Differential expression of glycan structures on the cell surface

**Fig 1:**
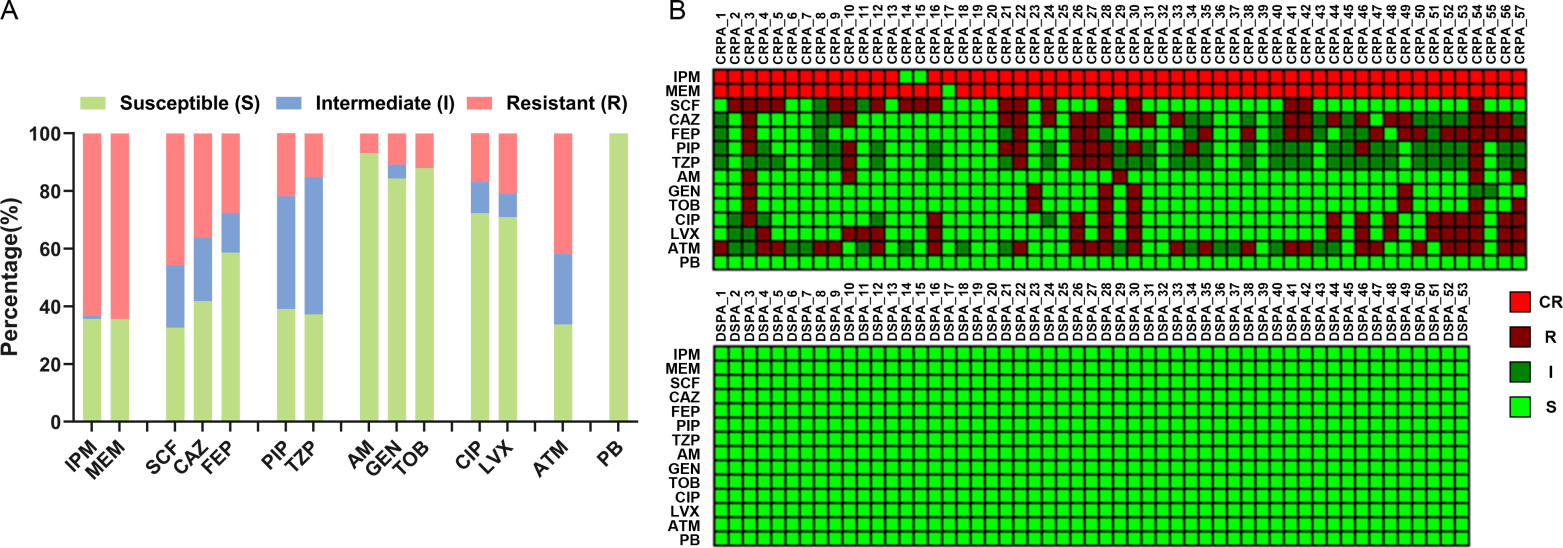
Strain characteristics of *P. aeruginosa* clinical isolates. (**A**) Percent stacked bar-plot of antibiotic resistance ratio among 87 MDR *P. aeruginosa* isolates. (**B**) Heat map of antibiotic susceptibility patterns of 57 CRPA and 53 DSPA isolates. CR, carbapenem-resistant; R, resistant; I, intermediate; S, susceptible; IPM, imipenem; MEM, meropenem; SCF, cefoperazone/sulbactam; CAZ, ceftazidime; FEP, cefepime; PIP, piperacillin; TZP, piperacillin/tazobactam; AM, amikacin; GEN, gentamicin; TOB, tobramycin; CIP, ciprofloxacin; LVX, levofloxacin; ATM, aztreonam; and PB, polymyxin B.

Imipenem (IPM) and meropenem (MEM) are members of carbapenems and are routinely used to treat *P. aeruginosa* infections (44). In the present study, *P. aeruginosa* tested in the clinical laboratory for resistance to IPM and/or MEM was defined as CRPA. A total of 140 isolates from the clinic were analyzed for antimicrobial susceptibility, of which 53 isolates (37.86%) were susceptible to antimicrobial agents and 87 isolates (62.14%) were resistant strains. A large proportion of these resistant isolates showed high-level resistance to carbapenems (Fig. 1A), with 63.22% resistance to IPM and 64.37% resistance to MEM, and a total of 57 strains were CRPA, almost all of which were resistant to both IPM and MEM (Fig. 1B).

**Fig 2:**
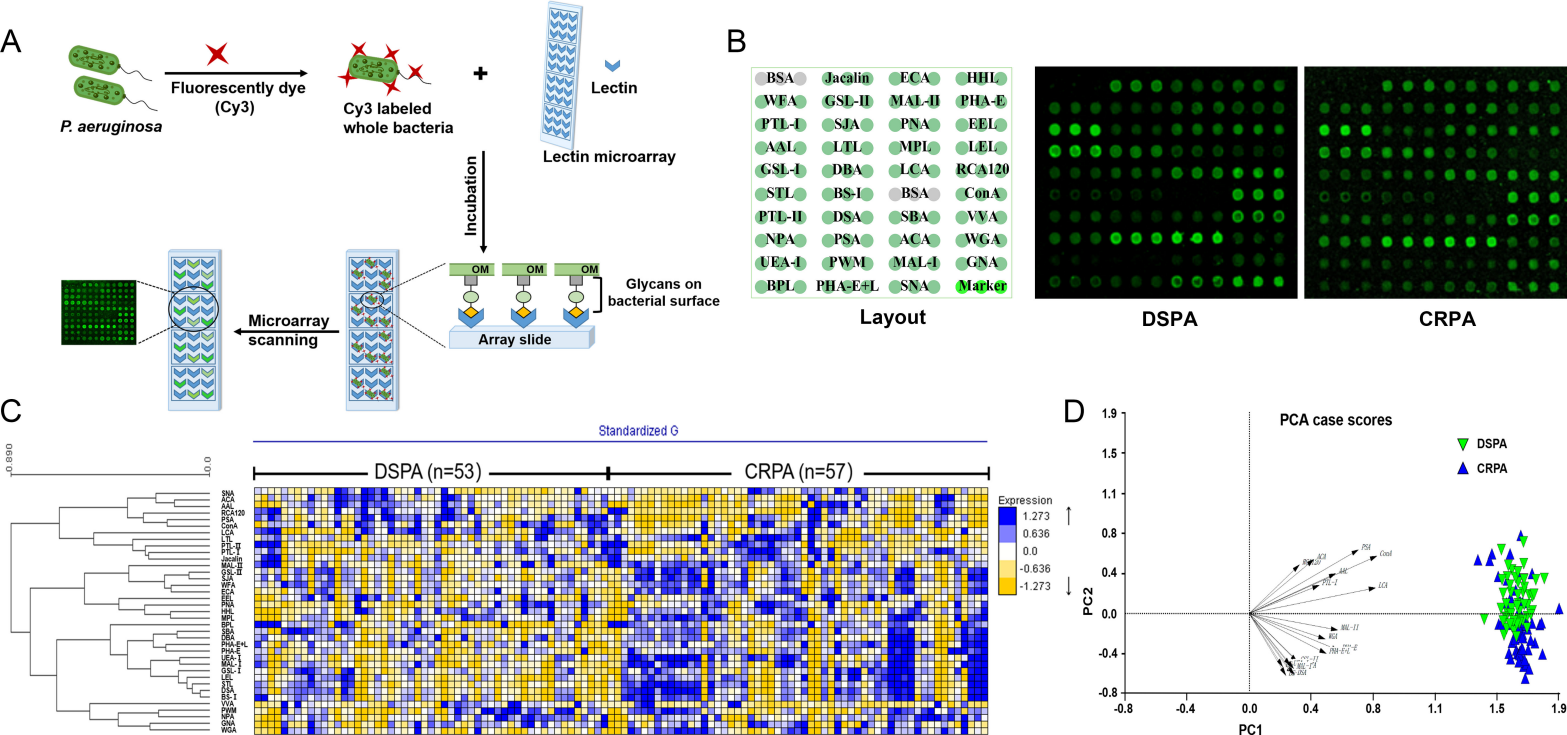
Lectin microarray analysis of whole bacteria. (**A**) The process diagram of Cy3-labeled whole bacteria binding to lectin microarray through the interaction between bacterial surface glycans and lectins. (**B**) (Left) Lectin microarray format. Glycan-binding specificities of the lectins are listed in Table 1. (Right) Glycopatterns of bacterial surface of DSPA and CRPA according to lectin microarray. A representative microarray scanning image was selected from each group. (**C**) Heat map of comparison of lectin microarray data for 53 DSPA and 57 CRPA isolates. The levels of lectin binding signals were indicated by the color change from yellow (low binding level) to blue (high binding level). (**D**) Principal component analysis of 20 lectins microarray data with relative changes in DSPA and CRPA groups.

**Fig 3:**
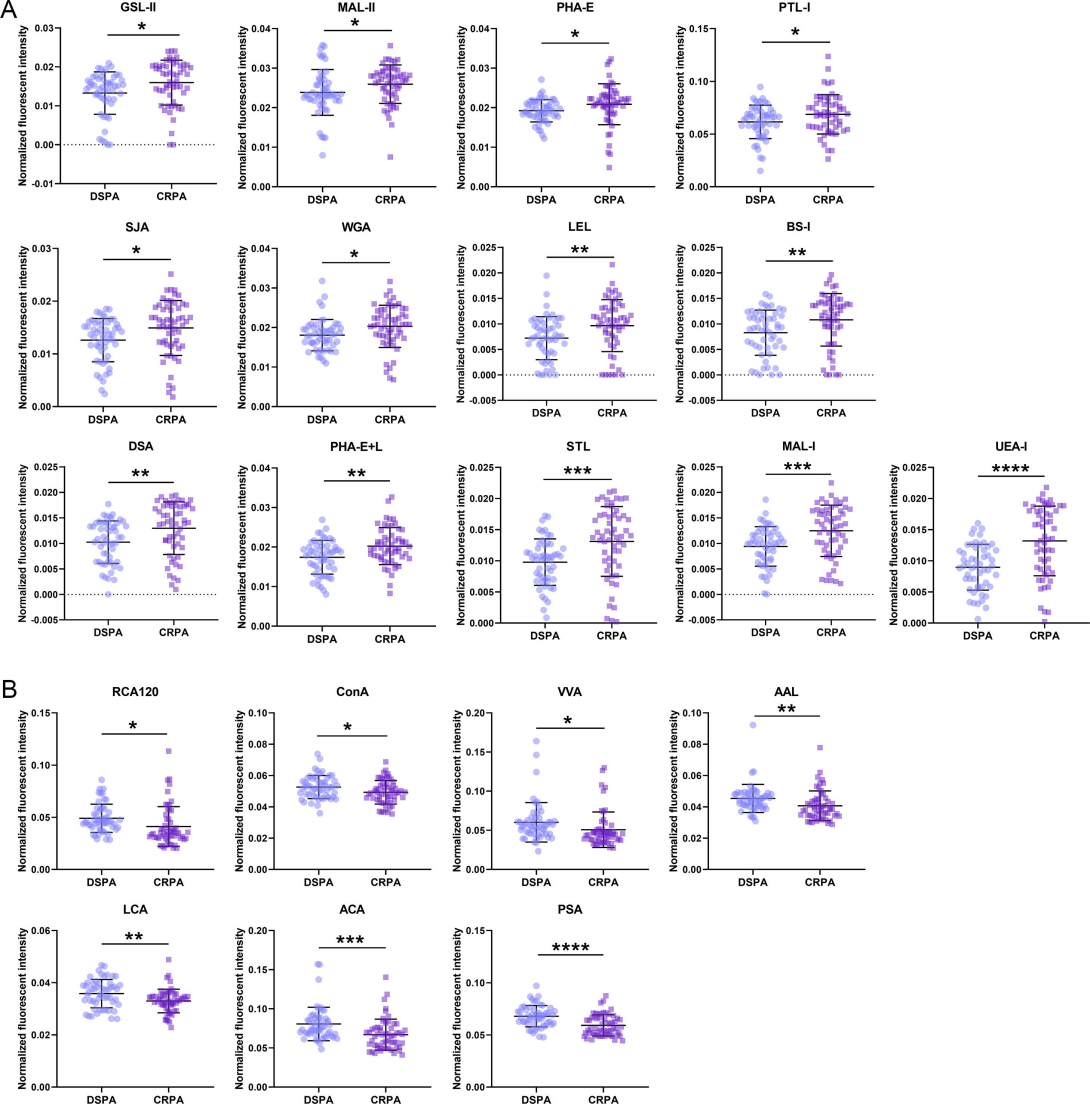
Comparison of glycan expression on the bacterial surface between DSPA and CRPA. (**A and B**) Glycan expression is up-regulated (**A**) and down-regulated (**B**) in CRPA, respectively. Statistical significance was calculated using an unpaired two-tailed *t*-test; *P < 0.05; **P < 0.01; ***P < 0.001; ****P < 0.0001. Error bars represent standard deviation.

To determine whether and which association exists between carbapenem resistance and glycan structures, we first examined differences in the expression levels of glycan structures on the bacterial cell surface between DSPA and CRPA strains using lectin microarrays. The abundance of carbohydrates and glycoconjugates distributed on the bacterial cell surface allowed the whole bacteria fluorescently labeled with Cy3 dyes to bind to the microarrays through the interaction of cell surface glycan with lectin on a glass slide (28, 40) (Fig. 2A). One hundred and ten samples of bacterial strains (DSPA = 53, CRPA = 57) were examined independently to obtain the corresponding microarray fluorescence signal (Fig. 2B). After normalized fluorescence intensities (NFIs), a heat map was created using hierarchical clustering to visualize the lectin signal intensity distribution (Fig. 2C), confirming significant differences in bacterial surface glycan expression between DSPA and CRPA. Further between-group comparisons of each of the 37 lectins were performed. The relationship between DSPA and CRPA was visualized by principal component analysis (PCA) (Fig. 2D), which was performed on the data generated collectively for the NFIs of the 20 altered lectins in the 110 samples. The PCA results displayed a tendency for the subjects assigned to the scatterplot to cluster separately to form DSPA and CRPA pools. The presence of the partial non-overlapping region between the two pools suggests that it is possible to differentiate between DSPA and CRPA based on specific variations in bacterial glycopatterns. Compared to the DSPA group, 13 lectins of GSL-II, MAL-II, PHA-E, PTL-I, SJA, WGA, LEL, BS-I, DSA, PHA-E+L, STL, MAL-I, and UEA-I showed increased changes of NFIs in CRPA group (Fig. 3A), and among these lectins, high mannose-type *N*-glycans recognized by LEL; Galactose recognized by BS-I; GlcNAc recognized by DSA, PHA-E+L, and STL; Galβ1–4GlcNAc and Galβ1–3GlcNAc recognized by MAL-I; and Fucα1–2Galβ1–4Glc(NAc) recognized by UEA-I increased significantly (*P* value < 0.01); the NFIs of 7 lectins, RCA120, ConA, VVA, AAL, LCA, ACA, and PSA, were decreased in CRPA group (Fig. 3B), where the Fucα1–6GlcNAc (core fucose) and Fucα1–3(Galβ1–4)GlcNAc recognized by AAL, α-d-Man and Fucα1–6GlcNAc recognized by LCA and PSA, and Galβ1–3GalNAcα-Ser/Thr(Tn) recognized by ACA were significantly reduced (*P* value < 0.01) (Table 1). These results show that the expression levels of multiple glycan structures on the bacterial surface were altered between the DSPA and CRPA groups.

**TABLE 1:** Lectin information contained on the microarray

Abbreviation	Lectin	Specificity	Containing monosaccharide^*a*^	Two-tailed *t* test (*P* value)
				DSPA vs CRPA
**Jacalin**	*Artocarpus integrifolia* lectin	Galβ1–3GalNAcα-Ser/Thr(T), GalNAcα-Ser/Thr(Tn)	Galactose	0.5021, ns
**ECA**	*Erythrina cristagalli* lectin	Galβ1–4GlcNAc (type II), Galβ1–3GlcNAc (type I)	Galactose	0.1146, ns
**HHL**	*Hippeastrum* hybrid lectin	α1–3/1–6Man, Galα1–3Galβ1–4GlcNAc	Mannose	0.6192, ns
**WFA**	*Wisteria floribunda* lectin	GalNAcα/β1–3/6Gal	GalNAc	0.6036, ns
**GSL-II**	*Griffonia simplicifolia* lectin II	GlcNAc and agalactosylated tri/tetra antennary glycans	GlcNAc	0.0139, *
**MAL-II**	*Maackia amurensis* lectin II	Siaα2–3Galβ1–3GalNAc, Siaα2–3Galβ1–4Glc (NAc)/Glc, Siaα2–3Gal, Siaα2–3, Siaα2–3GalNAc	–	0.0042, *
**PHA-E**	*Phaseolus vulgaris* erythroagglutinin	Galβ1–4GlcNAc, biantennary *N*-glycan	GlcNAc	0.0042, *
**PTL-I**	*Psophocarpus tetragonolobus* lectin I	GalNAc, GalNAcα1–3Gal, GalNAcα1–3Galβ1-3/1-4Glc	GalNAc	0.0037, *
**SJA**	*Sophora japonica* agglutinin	Terminal GalNAc/Gal	GalNAc	0.0115, *
**PNA**	Peanut agglutinin	Galβ1–3GalNAcα-Ser/Thr(T)	Galactose	0.9500, ns
**EEL**	*Euonymus europaeus* lectin	Galα1–3(Fucα1–2)Gal	Galactose	0.5757, ns
**AAL**	*Aleuria aurantia* lectin	Fucα1–6GlcNAc (core fucose), Fucα1–3(Galβ1–4)GlcNAc	Fucose	0.0088, **
**LTL**	*Lotus tetragonolobus* lectin	Fucα1–2Galβ1–4GlcNAc, Fucα1–3(Galβ1–4)GlcNAc	Fucose	0.2665, ns
**MPL**	*Maclura pomifera* lectin	Galβ1–3GalNAc, GalNAc	GalNAc	0.0641, ns
**LEL**	*Lycopersicon esculentum* lectin	(GlcNAc)n, high mannose-type *N*-glycans	LacNAc	0.0072, **
**GSL-I**	*Griffonia simplicifolia* lectin I	αGal, αGalNAc	GalNAc	0.1731, ns
**DBA**	*Dolichos biflorus* agglutinin	GalNAc, GalNAcα1–3(Fucα1–2)Gal	GalNAc	0.5546, ns
**LCA**	*Lens culinaris* agglutinin	α-d-Man, Fucα1–6GlcNAc, α-d-Glc	Mannose	0.0036, **
**RCA120**	*Ricinus communis* agglutinin I	Gal, Galβ1–4GlcNAc, Galβ1–3GlcNAc	Galactose	0.0148, *
**STL**	*Solanum tuberosum* lectin	Core GlcNAc, oligosaccharide containing GlcNAc	GlcNAc	0.0004, ***
**BS-I**	*Bandeiraea simplicifolia* lectin I	α-Gal, α-GalNAc, Galα-1,3Gal, Galα-1,6Glc	Galactose	0.0072, **
**ConA**	*Concanavalin A*	High-Mannose, Manα1–6(Manα1–3)Man, terminal GlcNAc	Mannose	0.0206, *
**PTL-II**	*Psophocarpus tetragonolobus* lectin II	Gal	Galactose	0.9891, ns
**DSA**	*Datura stramonium* lectin	β-GlcNAc, Galβ1–4GlcNAc	GlcNAc	0.0029, **
**SBA**	Soybean agglutinin	α-/β-terminal GalNAc, (GalNAc)n	GalNAc	0.2134, ns
**VVA**	*Vicia villosa* lectin	Terminal GalNAc, GalNAcα-Ser/Thr(Tn), GalNAcα1–3Gal	GalNAc	0.0391, *
**NPA**	*Narcissus pseudonarcissus* lectin	High mannose	Mannose	0.9613, ns
**PSA**	*Pisum sativum* agglutinin	α-d-Man, Fucα1–6GlcNAc, α-d-Glc	Fucose	<0.0001, ****
**ACA**	*Amaranthus caudatus* lectin	Galβ1–3GalNAc	Galactose	0.0008, ***
**WGA**	Wheat germ agglutinin	Multivalent Sia and (GlcNAc)n	GlcNAc	0.0150, *
**UEA-I**	*Ulex europaeus* agglutinin I	Fucα1–2Galβ1–4Glc(NAc)	Fucose	<0.0001, ****
**PWM**	*Phytolacca americana* lectin	GlcNAc, Galβ1–4GlcNAc	GlcNAc	0.6568, ns
**MAL-I**	*Maackia amurensis* lectin I	Galβ1–4GlcNAc, Galβ1–3GlcNAc	Galactose	0.0005, ***
**GNA**	*Galanthus nivalis* lectin	High-Mannose, Manα1–3Man	Mannose	0.6600, ns
**BPL**	*Bauhinia purpurea* lectin	Galβ1–3GalNAc, Terminal GalNAc	Galactose	0.1182, ns
**PHA-E+L**	*Phaseolus vulgaris* agglutinin	Bisecting GlcNAc, bi-antennary *N*-glycans, tri- and tetra-antennary complex-type *N*-glycan	GlcNAc	0.0013, **
**SNA**	*Sambucus nigra* lectin	Siaα2–6Gal/GalNAc	GlcNAc	0.7945, ns

^*a*^ The monosaccharide that should be added to the lectin solution before printing. Detailed information can also be found in the information provided by the manufacturer of the lectin. Fuc, fucose; Man, mannose; Gal, galactose; Glc, glucose; Sia, sialic acid; GlcNAc, N-acetyl-glucosamine; GalNAc, N-acetyl-galactosamine; LacNAc, N-acetyl-lactosamine; Ser, serine; Thr, threonine; Tn, Tn antigen. P value: *, *P* < 0.05; **, *P* < 0.01; ***, *P* < 0.001; ****, *P* < 0.0001; ns, not significant.

### Differential expression of glycan structures in the intracellular space

**Fig 4:**
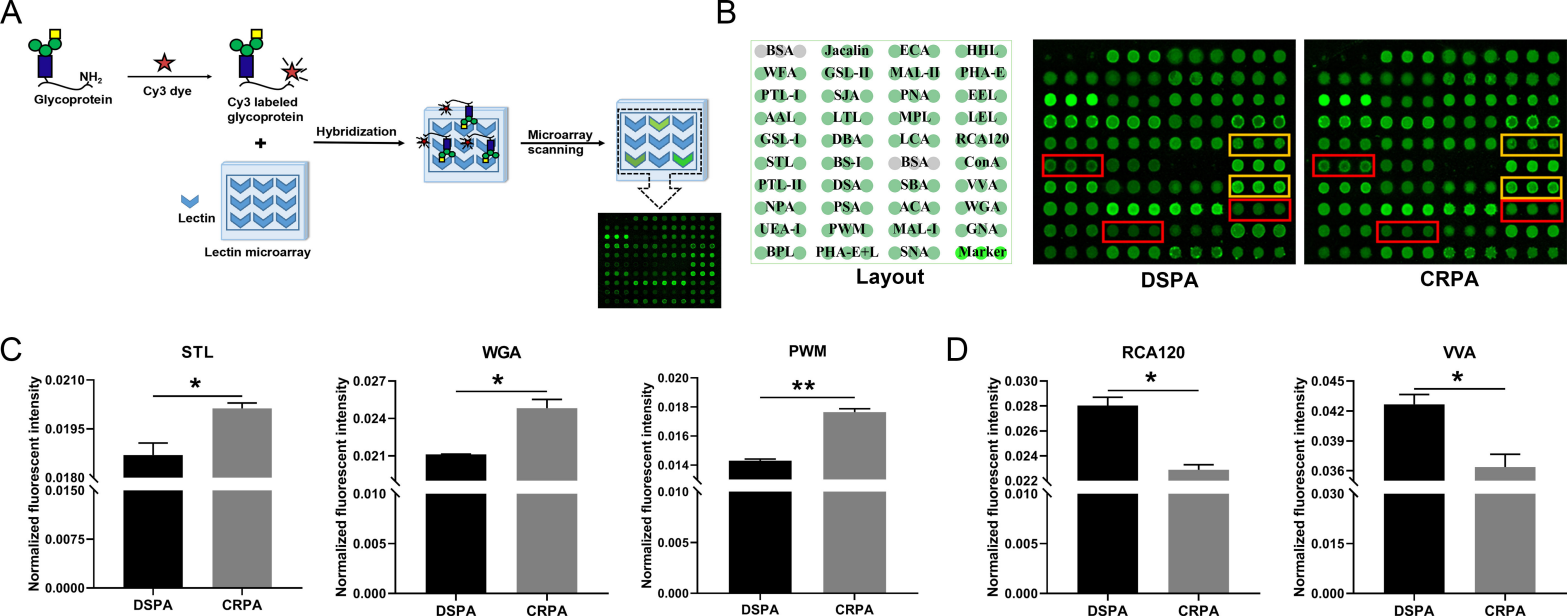
Lectin microarray analysis of bacterial intracellular proteins. (**A**) The basic procedures of glycoprotein bind to the lectin microarray. (**B**) Glycopatterns of lectin microarrays of intracellular protein mixtures from the DSPA and CRPA groups. Red boxes marked on the microarrays indicate increased expression and yellow boxes indicate reduced expression. (**C and D**) Glycan expression is up-regulated (**C**) and down-regulated (**D**) in CRPA, respectively. Data shown are means ± SEM; *P < 0.05, **P < 0.01 (unpaired two-tailed *t*-test, *n* = 2).

In bacteria, glycoproteins are located in diverse cellular compartments, ranging from the cell wall and cytoplasmic membrane proteins to structural components of ribosomes (27). Whether the *en bloc* or a sequential addition glycosylation pathway, the abundant presence of glycoconjugates on the bacterial cell surface starts in the cytoplasm (nucleotide sugars), subsequently undergoes synthesis (or even extension) and is eventually transferred to the periplasm or outer membrane (10, 13). Whole-bacteria analysis relies on the direct and specific recognition and binding of glycan structures on the bacterial cell surface to lectins on microarrays to capture bacteria onto glass slides, and the intensity of the collected fluorescent signals reflects the expression level of the glycan structures on the bacterial surface. Changes in intracellular glycan structures are not available without a separate detection. The abundant glycan structures loaded on the bacterial surface originate from various intracellular glycosylation processes and their involved elements. To systematically detect changes in the glycan structures of entire bacteria, as well as to highlight the abundance of changes on the bacterial surface, it is also necessary to clarify whether the expression level of intracellular glycan structures has been changed. Therefore, we disrupted the bacterial cell wall by ultrasound to obtain intracellular soluble components (proteins, nucleic acids, etc.), and the determination of intracellular glycan changes proceeded as shown in Fig. 4A. Before fluorescent labeling, we pooled supernatants from each of the 53 DSPA strains and 57 CRPA strains in equal amounts to a final volume of 2 mL to perform microarray analysis. The layout of the lectin recognition pattern of Cy3-labeled pooled DSPA sample and pooled CRPA sample is shown in Fig. 4B. Statistical analysis of the NFIs for each lectin showed that in CRPA, the NFIs for three lectins, STL, WGA, and PWM, which recognize GlcNAc were significantly increased (Fig. 4C); the NFIs for two lectins, Galactose and GalNAc recognized by RCA120, GalNAc and GalNAcα-Ser/Thr(Tn) recognized by VVA, were significantly reduced (Fig. 4D). The other 32 of the 37 lectins showed no signal differences between DSPA and CRPA, indicating that alterations in intracellular glycan structures are relatively constant. Based on the above comparisons, we will follow by focusing on the changes in glycan structures on the bacterial surface.

### Validation of the differential expression of glycan structures

**Fig 5:**
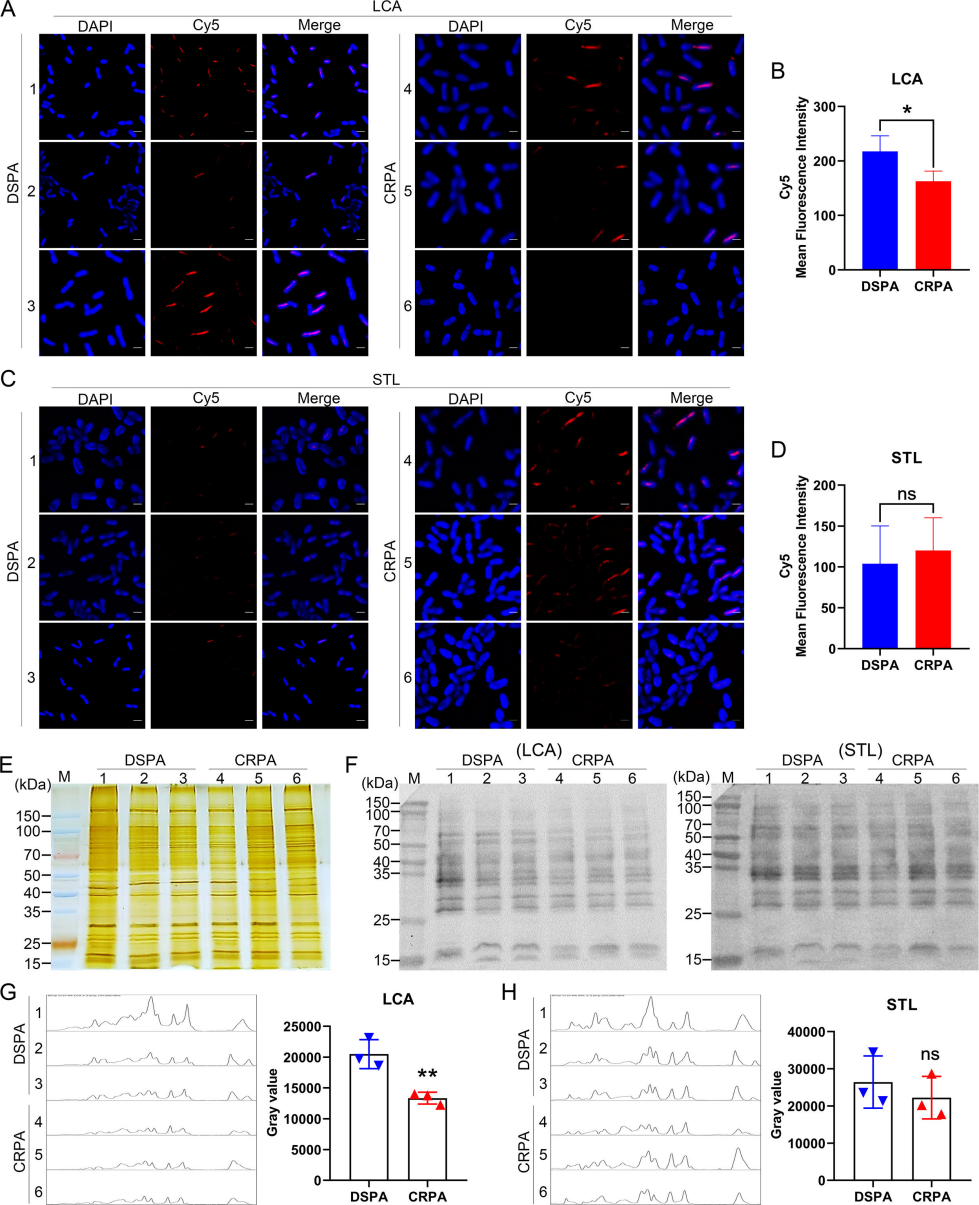
Validation of the differential expression of glycan structures on the cell surface. (**A and C**) Immunofluorescence analysis of individual strains between DSPA and CRPA. Representative images of whole bacteria stained with DAPI (blue) and hybridized with Cy5-labeled lectins [LCA (**A**) and STL (**C**)] (Cy5, red). Scale bars: 2 µm. (**B and D**) Quantification of the mean fluorescence intensity of lectin-bound [LCA (**B**) and STL (**D**)] bacterial cells with Cy5 foci. Statistical significance was determined using a two-tailed unpaired *t*-test; *P < 0.05; ns, not significant. (**E**) Silver-stained SDS-PAGE of whole-bacterial proteins obtained from six selected isolates. M, Protein marker. (**F**) The binding pattern of glycoproteins in selected samples of DSPA and CRPA isolates was analyzed by using two lectins [LCA (left) and STL (right)]. (**G and H**) Gray-value plots for each lane of LCA (**G**) and STL (**H**) and quantification of gray value by image J. Statistical significance was determined using a two-tailed unpaired *t*-test; **P < 0.01; ns, not significant. (**A–H**) 1–3: three DSPA strains (DSPA_1, 2, 3); 4–6: three CRPA strains (CRPA_6, 13, 20). Similar results were obtained from three independent experiments, and the data shown are from one representative experiment.

To validate the differential expression of glycan structures on the bacterial surface, we performed lectin-based immunofluorescence analysis with LCA and STL between 3 DSPA and 3 CRPA (samples selected randomly from each group). Cy5-labeled LCA and STL were incubated with whole bacteria, respectively, and the expression levels of specific glycan structures on the cell surface were characterized by their interactions with the lectins. Immunofluorescence signals were visualized using confocal microscopy (Fig. 5A and C) and quantified in selected areas by the mean gray value (equal to the ratio of integrated density to the area) (Fig. 5B and D). The results showed that the expression level of glycan structures recognized by LCA was down-regulated in the CRPA group compared to the DSPA group (Fig. 5A and B), which was consistent with the results of the lectin microarrays; there was no significant difference in the expression of glycan structures recognized by STL (Fig. 5C and D), which failed to align with the significant up-regulation detected by the lectin microarrays. This means that changes of glycan structures recognized by certain lectins (such as STL) obtained using lectin microarrays may be relative changes after normalizing fluorescence intensities. To further confirm the results, we implemented lectin blotting to analyze the binding of LCA and STL with glycan structures on whole bacterial proteins, respectively. Silver staining showed that the molecular weight and global abundance of proteins were similar in the six isolates although with several distinct bands (40–70 kDa) (Fig. 5E). Lectin blots showed the patterns of proteins loading with glycan structures bound to LCA or STL (Fig. 5F). For clarity, the results of lectin blots were quantified by the total gray values of the bands in each lane of the six selected strains to compare the differences between 3 DSPA and 3 CRPA, which showed that the expression of glycan structures bound by LCA was decreased in the CRPA group (Fig. 5G), whereas the expression of glycan structures bound by STL was not significantly different between the two groups (Fig. 5H), which was consistent with the results of the immunofluorescence analysis. LCA and STL were randomly selected as representatives to validate the results of the lectin microarrays; the validation for the remaining changed lectins was not shown here.

### Selection of CRPA-related characteristic glycans and construction of machine-learning model using GBDT algorithm

**Fig 6:**
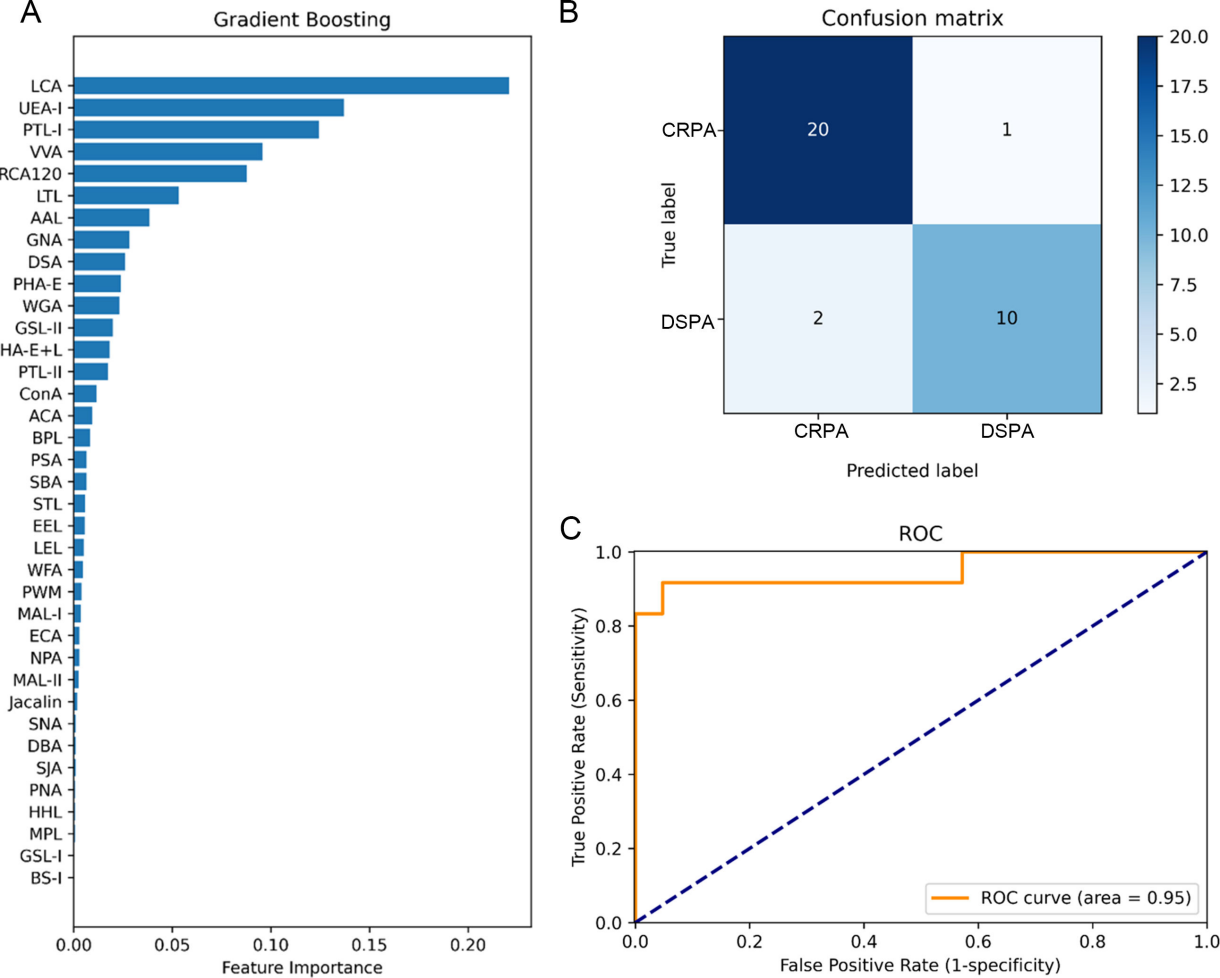
Performance of the GBDT model. (**A**) Feature importance ranking of GBDT. GBDT, gradient boosting decision tree. (**B**) Confusion matrix of the model. The row of the confusion matrix represents the actual value in the data set, and the column represents the predicted label of the classifier. (**C**) Model receiver operating characteristic (ROC) curves.

GBDT is an integrated algorithm based on a decision tree that performs well in data analysis and prediction (45 – 47). To achieve data availability of the microarray assay and to identify potential features of glycan-based alterations associated with CRPA, we constructed a model for differentiating DSPA and CRPA strains through machine learning based on the GBDT algorithm. It is fruitful to screen for major glycan features associated with carbapenem resistance during the establishment of the classification model. As shown in Fig. 6A, the GBDT classifier ranked the importance of the individual variables according to their relative influence, with LCA (recognizing α-d-Man and Fucα1–6GlcNAc) as the top weighting feature. The top 5 important features also included UEA-I [recognizing Fucα1–2Galβ1–4Glc(NAc)], PTL-I (recognizing GalNAcα1–3Gal and GalNAcα1–3Galβ1-3/4Glc), VVA [recognizing GalNAc and GalNAcα-Ser/Thr(Tn)], and RCA120 (recognizing Galβ1–4GlcNAc and Galβ1–3GlcNAc) (Fig. 6A).

In an independent validation cohort of 33 isolates, the classifier correctly predicted 11 strains out of 12 DSPA and 21 strains out of 21 CRPA (Fig. 6B), presenting the high efficiency of the classifier. The performance of the test set was calculated with an AUROC of 0.95 (Fig. 6C), average classification precision of 89.29%, average recall of 90.9%, accuracy of 91%, and F1 score of 0.91 (Table 2). Sensitivity and specificity were 0.917 and 0.952, respectively. These exhibited the good performance of the feature-based classification model, which means that the glycopatterns such as α-d-Man and Fucα1–6GlcNAc recognized by LCA can be used as biomarkers for the differentiation of clinical DSPA and CRPA.

**TABLE 2:** The performance of GBDT model

	Precision	Recall	F1-score	Support
**CRPA**	0.91	0.95	0.93	21
**DSPA**	0.91	0.83	0.87	12
**Accuracy**	0.89	0.91	0.91	33
**Macro avg**	0.91	0.89	0.90	33
**Weighted avg**	0.91	0.91	0.91	33

## DISCUSSION

The resistance mechanisms identified in carbapenem-resistant *P. aeruginosa* are generally the loss of the expression of the outer membrane protein OprD and overexpression of the efflux pumps (MexAB-OprM and MexCD-OprJ), the expression of intracellular β-lactamases, and the acquisition of multiple carbapenemase genes, etc. (34); however, these well-defined mechanisms have not yet been effective in solving the therapeutic challenges of infections with the clinical strains. Clinical isolates often carry multiple resistance mechanisms simultaneously. It is more clinically realistic to consider the co-existence of multiple resistance mechanisms when controlling infections caused by resistant isolates. It should be perceived that the same resistance phenotype is a common feature of resistant isolates and distinguishes them from sensitive strains. In this work, we initiated a preliminary speculation as to whether clinical isolates with the same resistance phenotype would show identical glycan structures changes. Lectin microarrays were employed to compare the expression levels of glycan structures between DSPA and CRPA of clinical isolates. It was found that the glycan changes occurring on the bacterial surface were significantly abundant (Fig. 2 and 4). This is likely because the majority of bacterial carbohydrates are distributed on the bacterial surface, which acts as a direct and permeable barrier to antibiotic pressure. Of the 37 lectins we selected for fixation on microarrays, 20 lectins with alterations in NFIs contained recognition of the glycan structures of GlcNAc, Fucose, Galactose, GalNAc, LacNAc, and Mannose (Fig. 3; Table 1). The results predict the possibility of the presence of multiple glycosylation systems associated with antibiotic resistance, but the mechanisms involved have yet to be supported by sufficient and detailed evidence. Cluster analysis showed that there were differences in glycopatterns between individual isolates (Fig. 2C), and such respective differences might be related to resistance mechanisms, genotypes, or carrying resistance to other antibiotics. However, the PCA displayed the partial non-overlapping region that represents clustering separately to form DSPA and CRPA pools (Fig. 2D), suggesting that it is possible to differentiate between DSPA and CRPA based on glycopatterns. Immunofluorescence analysis and lectin blotting validated the results of lectin microarrays for two randomly selected lectins (LCA and STL) in 3 DSPA and 3 CRPA (Fig. 5). The validation results for LCA were in agreement with the results of the lectin microarrays, whereas the results for STL were inconsistent. As shown in Fig. 3A, the scatter plot for STL showed relatively discrete clustering of sample scatters. The likely reason is that the microarrays assay involves the competitive recognition and binding of 37 lectins, while the validation analysis involves the contact of individual lectins with the glycan structures on the bacterial surface without competition between lectins, indicating the occurrence of relative alterations when normalized fluorescence intensities for all lectins on glass slide. Therefore, LCA was an absolute variable involved in the differential expression of glycan structures on the bacterial surface between DSPA with CRPA, whereas the glycan structures recognized by STL may not altered. On the other hand, small sample sizes may also lead to inconsistent validation results due to the large differences in subtypes between clinical strains. To further screen for important glycopatterns capable of distinguishing carbapenem resistance, machine learning was then implemented to construct a classifier model. Our results illustrate a series of good performance parameters for the predicted model with lectins such as LCA and UEA-I as the important features (Fig. 6; Table 2). In turn, the model performance reflects the fact that glycan structures recognized by the lectins can be used as biomarkers to distinguish clinical CRPA from DSPA.

Several studies have found that *P. aeruginosa* isolated from hospital environments exhibits high genotypic diversity and broad antimicrobial resistance spectra. Similarly, the isolates we collected also exhibited MDR to various antibiotics, with the highest ratios of resistance to carbapenems (Fig. 1A and B). Therefore, analyzing only a single isolate may be confounded by genotypic and multiple resistance, resulting in reduced clinical significance. For this reason, we analyzed a total of 110 clinical isolates from a hospital, divided into a DSPA group and a CRPA group, and we believe that the analysis of multiple samples in each group is more relevant for clinical guidance. This is also inspired by the widespread application of lectin microarray to assess tumor characteristics and screen new biomarkers for diagnostic cancer (48 – 52). When a large number of samples are collected within each group, the changes in expression levels of glycan structures sought are more representative of the various pathological and physiological states, as a clinical disease is often accompanied by other conditions, such as different complications between individuals with diabetes. Data from the microarray showed that glycopatterns differed between strains of different subtypes (Fig. 2C), but that the CRPA group exactly possessed significant glycan alterations that differed from the DSPA group (Fig. 2C and D), an exciting starting point for discovering the glycopatterns as biomarkers associated with antibiotic resistance.

The development of artificial intelligence as machine learning has shown prospects in the evaluation of disease-based diagnostic efficacy (53, 54). We follow the idea of differentiating clinical disease by identifying biomarkers through models that look for potential markers associated with CRPA, an approach that has shown credible accuracy and specificity when evaluating diagnostic efficacy through model construction (55). In this study, we constructed a GBDT-based machine-learning model to differentiate between DSPA and CRPA. During the construction process, α-d-Man and Fucα1–6GlcNAc recognized by LCA were found to be the most important variables that can differentiate between clinical DSPA and CRPA with good performance (Fig. 6). The representational glycan structures identified through this method are extremely reliable as biomarkers for CRPA.

Analysis of glycan structures on the bacterial surface realistically reflects the transformation in glycan signaling associated with clinical infectious *P. aeruginosa* under different selective pressures of antibiotics. The known monosaccharides structure on *P. aeruginosa* surface includes GlcNAc, GalNAc, ManNAc, FucNAc, and other unique monosaccharides (e.g., Xyl, Kdo, Rha, etc.) (8, 56). The association between these monosaccharide-related glycan structures and antimicrobial resistance in *P. aeruginosa* has not been clearly described. *C. jejuni* is a well-studied model organism for bacterial glycosylation. The *N*-oligosaccharyltransferase PglB is the key enzyme that catalyzes *N*-glycosylation of, at least, 53 proteins in *C. jejuni* (57, 58). Disruption of *pglB* ablates the glycosylation of CmeABC, resulting in impairment of the efflux activity of CmeABC, which reduces resistance to four different antibiotic classes (ampicillin, erythromycin, tetracycline, and ciprofloxacin) (59). Abouelhadid et al. showed that the loss of the *N*-linked glycans in CmeABC is the sole reason for the multidrug efflux pump impairment phenotype and not a pleiotropic effect caused by the disruption of the *N*-oligosaccharyltransferase PglB (37). Interestingly, *P. aeruginosa* also possesses the gene *pgl*, which was characterized as 6-phosphogluconolactonase. In most cases, the genes responsible for glycosylation are located in close vicinity of the gene encoding the target protein (60). In *P. aeruginosa* PAO1, the *pgl* is located adjacent to the glucose/carbohydrate outer membrane porin OprB (https://www.pseudomonas.com/). The Pgl responsible for the glycosylation of OprB remains unclear. Mutant strains lacking OprB have not been reported to be associated with carbapenem resistance and the porin protein associated with carbapenem resistance in *P. aeruginosa* is usually OprD (34). It remains to be elucidated which glycosylases regulate the glycosylation of OprD and whether this results in changes in antimicrobial resistance. Furthermore, if the function of Pgl in *P. aeruginosa* also affects the activity of the multidrug efflux pump, it is also possible that resistance to carbapenems could be affected. In contrast to the above thinking, the significant reduction in the expression level of Fucose-associated glycan structures recognized by LCA occurred on the bacterial surface, while the presence of Fucose in *P. aeruginosa* has not been reported. Fucosyltransferases (FucTs) are important tools for the synthesis of fucosylated glycoconjugates. The understanding of the fucosylation process and related FucTs in bacteria is also quite limited (61, 62) (http://www.cazy.org/bP.html). The presence of FucTs in clinical *P. aeruginosa* isolates is highly questionable because many pathogens can evolve to manufacture host-like carbohydrates on their cell surfaces to mimic host-promoting survival and infection behaviors (40, 63, 64). *P. aeruginosa* is highly adapted to its environment. In a host microenvironment receiving antibiotic treatment, bacteria may also be able to perform molecular mimicry to harness host glycosylation resources in response to antibiotic pressure. Therefore, the significant changes in Fucose detected by microarrays may represent a characteristic simulation advantage of clinical strains isolated from human hosts, compared to laboratory strains. At the same time, it also illustrates that the contribution of glycans in biological processes is complex. Fortunately, the specific glycan structures recognized by lectins are well defined, and it is, therefore, promising that we used 37 lectins recognizing different glycan structures immobilized on microarrays to detect resistance-related changes in bacterial surface glycans.

As a preliminary investigation of the relevance of CRPA to glycopatterns, our study also has some limitations. For example, we used Cy3 to label the samples to make the assay consistent between the bacterial surface and intracellular space, and the final detection was mainly the changes of glycan structures on each part of the glycoprotein. To detect changes in LPS structure, enzymatic hydrolysis may be required before labeling to expose the reactive amino groups for binding to Cy3. Moreover, our microarray contained only 37 lectins, of which did not print CSL that bind to a Rha structure, and Rha was the main backbone structure of LPS of *P. aeruginosa*. Therefore, we lacked a complete analysis of the LPS structure.

A series of detailed analysis and the implementation of machine learning have made the data obtained by microarray available and finally approached important glycopatterns for CRPA. Future studies of a clear mechanism will focus on these important glycan structures to find new targets for therapeutic intervention and novel glycol-antimicrobial agents to alleviate the fatal threat posed by clinical MDR *P. aeruginosa*. In conclusion, the application of lectin microarrays to explore glycan biomarkers associated with clinical bacterial resistance is feasible and it is promising to employ this technology to further understand the mechanism of the effects of antibiotic resistance and glycosylation.

## MATERIALS AND METHODS

### Bacterial strains and susceptibility assays

The strains used in this study were collected from clinical *P. aeruginosa* isolates isolated from the Department of clinical laboratory, No. 215 Hospital of Nuclear Industry in Shaanxi Province from December 2019 to June 2021. A total of 140 isolates were obtained from clinical samples, including sputum (75%), skin secretions (13%), and others of peritoneal drainage, whole blood culture, bile, urine, pleural effusion, cerebrospinal fluid, and catheters (12% in total). The clinical laboratory performed an identification of the strains using a fully automated bacterial analyzer such as VITEK 2 System (BbioMérieux), and the antibiotic susceptibility of the strains with 14 antimicrobial drugs of Carbapenems (Imipenem, Meropenem), Cephalosporins (Cefoperazone/Sulbactam, Ceftazidime, Cefepime), Penicillins (Piperacillin, Piperacillin/tazobactam), Aminoglycosides (Amikacin, Gentamicin, Tobramycin), Quinolones (Ciprofloxacin, Levofloxacin), Monocyclic β-lactams (Aminotransomide), Peptides (Polymyxin B) was presented by MIC values, with the VITEK 2 AST-GN09 card for the identification of Gram-negative bacilli. The interpretation of MICs was divided into three categories according to the Clinical Laboratory Standard Institute (CLSI) guidelines: antibiotic sensitivity, intermediate, and resistance. One hundred and ten isolates (DSPA = 53 and CRPA = 57) were included in a series of analyses of this study (Fig. 1B).

### Bacterial sample preparation and fluorescent labeling

Cells were harvested from bacterial cultures during the mid-exponential phase of growth at an OD_600_ of 1.0. The collected bacterial precipitate was washed 3 times with an equal volume of phosphate-buffered saline (PBS) by centrifugation at 6,000 × *g* for 5 min at 4°C.

For the preparation of intracellular protein samples, the bacterial cells were first resuspended in 2 mL PBS and added with 1% phenylmethylsulfonyl fluoride. Following sonication, cells were ruptured and then centrifuged at 8,000 × *g* for 10 min at 4°C to separate unbroken cells and any insoluble debris. The supernatant was filtered twice through a 0.22-µm filter to completely remove cell debris to obtain a clarified protein extract. One hundred microliters of prepared protein samples was extracted into 100 µL of 0.1M Na_2_CO_3_ (pH 9.3) solution and added 5 µL of 100 mg/mL activated Cy3 fluorescent dye. The mixture was incubated at 100 rpm for 0.5 h at 4°C, protected from the light. Next, 20 µL of 4 M hydroxylamine hydrochloride solution was added and reacted on ice in the dark for 20 min to terminate the covalent binding between the Cy3 and the amino group on the protein. The fluorescent-labeled protein was separated and purified using the Sephadex G-25 column (GE Healthcare Life Sciences). The protein concentration was quantified using BCA assay, and samples were stored at −20°C until used.

Differently, for the fluorescent labeling of the whole bacteria, we resuspended the bacteria with 200 µL of 0.1 M Na_2_CO_3_ (pH 9.3) solution, added 20 µL of 100 mg/mL activated Cy3 fluorescent dye, and incubated at 80 rpm for 1 h at room temperature protected from light. After the Na_2_CO_3_ solution containing the free Cy3 dye was removed by centrifugation at 3,000 × *g* for 5 min, the fluorescently labeled bacterial precipitate was collected. The bacteria were washed five times with 800 µL PBS to remove the unbound Cy3 dye wrapped between the bacterial precipitates. The fluorescently labeled bacterial cells were then resuspended in 100 µL PBS and temporarily stored at 4°C protected from the light.

### Lectin microarray analysis

Lectin microarray analysis was performed as previously described (65) with minor modifications. Briefly, 37 different lectins were immobilized on slides modified by epoxidation. Each lectin was printed in triplicate spots in four replicate subarrays on each microarray slide. After ensuring that the lectin was firmly adhered to and stable on the chip surface, the lectin microarray was submerged into PBST buffer (10 mM PBS containing 0.2% Tween 20) and washed twice at 80 rpm for 5 min, and twice with PBS. Six hundred microliters of blocking buffer (10 mM PBS; 2% BSA; 500 mM glycine; 0.05% Tween 20) was added to the microarray hybridization cassette, covered with a cleaned lectin microarray and incubated in the microarray hybridization chamber for 1 h at 37°C protected from light. After the blocking step, the slides were washed twice with PBST and PBS, respectively.

Subsequently, for the analysis of bacterial intracellular protein samples, two micrograms of fluorescently labeled protein samples was mixed with incubation solution (10 mM PBS, 2% BSA, 500 mM glycine, 0.05% Tween 20, 10% 4 M hydroxylamine) and then added to a microarray hybridization cassette containing four subarrays (mixture system of 120 µL for each of microarray sectors), covered with a cleaned lectin microarray, and incubated in the microarray hybridization chamber for 1 h at 37°C protected from light. After incubation, the hybridized microarrays were washed, spin-dried, and scanned using an AXON GenePix (4100A) fluorescence microarray scanner to read the raw intensity values. For the detection of the whole bacteria, 40 µL of fluorescently labeled bacterial suspension was directly mixed with 90 µL of incubation solution. Then, 120 µL of the mixture was transferred to the microarray hybridization cassette according to the subarray region. The cleaned lectin microarray was covered similarly and incubated in the microarray hybridization chamber at 37°C for 3 h protected from light. After incubation, the hybridized microarrays were gently washed and air-dried in the dark. The final lectin microarray was scanned by AXON GenePix (4100A) scanner and the fluorescence signal value was obtained.

### Lectin-based immunofluorescence staining of whole bacteria and confocal microscopy

One hundred microliters of 1 mg/mL lectin solution was mixed with 100 µL of 0.1 M Na_2_CO_3_ solution (pH 9.3), and 5 µL of 100 mg/mL activated Cy5 fluorescent dye (GE Healthcare) was added to the mixed solution. The mixture was incubated in the dark at room temperature for 2 h. The incubated reaction was terminated by the addition of 20 µL of 4 M hydroxylamine hydrochloride on ice for 20 min. The fluorescent-labeled lectin was purified by Sephadex G-25 column. The concentration was determined by BCA assay, and the labeled lectins were stored at −20°C in darkness until use.

Bacteria were cultured as described above. One milliliter culture (OD_600_ of 1.0) was spun down for 5 min; 4°C; 8,000 rpm. Pellets were washed three times with precooled PBS at equal volume and then fixed in 750 µL of 4% precooled paraformaldehyde for 20 min at 4°C. Subsequently, bacteria were washed 3 times with 800 µL PBS and blocked with 1 mL Carbo-Free blocking solution for 1 h at room temperature. The blocked bacteria were then incubated with Cy5-labeled lectins (10 µg/mL in Carbo-Free blocking solution) with gentle shaking overnight at 4°C in the dark. The bacteria were washed 3 times with 800 µL PBS and then stained with 4,6-diamidine-2-phenylindol [DAPI (10 µg/mL), Sigma] for 5 min. Subsequently, bacteria were washed 2 times and resuspended with 200 µL PBS. The bacterial cell suspension (80 µL) was plated into a 15-mM glass bottom cell culture dish (NEST, 801002) and dried in the dark.

The Leica SP8 confocal microscope was used to capture the fluorescence signal of bacterial cells with a 63× oil objective, and the 638 nM (the Cy5 channel) and 405 nM (the DAPI channel) lasers were opened for fluorescence imaging. The bacterial fluorescence signal was quantitatively analyzed by Image J software.

### Silver staining and lectin blotting

Overnight cultures of bacteria were sub-cultured with 1% dilution in fresh LB medium and grown at 37°C with continuous shaking (220 rpm) to an OD_600_ of 1.0. The pelleted bacteria collected by centrifugation were resuspended in 100 µL of 1× SDS-loading buffer and incubated at 100°C for 20 min. Allow the solution to cool at room temperature for 15 min. Ten microliters of the prepared bacterial protein sample was separated by 12% SDS-PAGE gels and visualized using silver staining according to the standard protocol. In brief, the SDS gel was immersed in a fixative solution (50% ethanol, 10% glacial acetic acid) at room temperature for 1 h. Afterward, the gel was sensitized with sensitizing solution containing 0.2% sodium-thiosulfate for 30 min with gentle shaking and then washed four times with deionized water (DI water). The gel was stained with 1 g/L AgNO_3_ for 20 min and washed with DI water for 10–20 s. Carry out the color reaction for 5 min using a chromogen solution containing 35 g/L Na_2_CO_3_ and 0.1% formaldehyde. The reaction was terminated by a stop solution (10% glacial acetic acid). The gel was finally washed with DI water and imaged using a gel imaging system (Tanon, Shanghai, China).

For lectin blotting, the bacterial proteins in gels were transferred onto a polyvinylidene difluoride membrane and blocked with Carbo-Free blocking solution for 1 h at room temperature. The blocked membrane was then incubated with Cy5-labeled lectins (10 µg/mL in Carbo-Free blocking solution) with gentle shaking overnight at 4°C in the dark. The membranes were then washed twice each for 5 min with TBST and scanned by red fluorescence channel (635 nM excitation/650LP emission) with the voltage of 800 PMT using a phosphorimager (Storm 840, Molecular Dynamics). The gray values were calculated by Image J software.

### Construction of machine-learning model

In this study, 110 clinical *P. aeruginosa* isolates were divided into the DSPA group (53 strains) and CRPA group (57 strains). The data from the lectin microarray analysis of 110 strains were randomly split into a 70% training set and a 30% test set (66 – 69). We utilized the open-source Python machine-learning library sci-kit-learn to train and build a machine-learning model based on a GBDT algorithm. We applied fivefold cross-validation to determine the optimal parameters for the training set. With the optimal parameters tuned, 70% of lectin microarray data were used to train machine-learning algorithms to construct predictive models. Then, the performance of the established model was verified using 30% of the lectin microarray data. Calculation of the model performance measurement parameters was achieved in the test cohort with the help of feature importance analysis, confusion matrix, and receiver operating characteristic (ROC) curve analysis. The classification performance of the model was evaluated by calculating the F1 score, precision, recall, and support value (70).

### Data pre-processing and statistical analysis

After extracting the accurate microarray analysis data from the scanned images by GenePix Pro 6.0 Microarray Image Analysis Software, the raw data were subjected to normalization. The signal intensity value corresponding to each lectin spot is subtracted from the background signal intensity value of the spot as the effective signal value for each spot. Each lectin corresponds to three replicate spots, and the median of the effective signal values corresponding to the three replicate spots is taken so that each lectin in a subarray region corresponds to a median value. The proportion of the median value of each lectin to the sum of the median values of all the lectins was then calculated to perform the global normalization of the data, i.e., the normalized fluorescent intensities for each lectin. Tables were visualized in Microsoft Excel, and Cluster analysis was performed using Expander 8.0 software. Graphs were generated using GraphPad Prism 8.0 software and statistically analyzed by the *t*-test parametric test, with differences considered statistically significant at *P* value <0.05. In addition, the data were further analyzed by PCA.
